# The impact of dependence on advanced imaging techniques on the current radiology practice

**DOI:** 10.1016/j.amsu.2022.103708

**Published:** 2022-05-06

**Authors:** Summaiya Waheed, Muhammad Junaid Tahir, Irfan Ullah, Osamah Alwalid, Syeda Ghazala Irshad, Muhammad Sohaib Asghar, Zohaib Yousaf

**Affiliations:** a Dow Medical College, Dow University of Health Sciences, Karachi, Pakistan; b Dr Ruth K.M. Pfau, Civil Hospital, Karachi, Pakistan; c Lahore General Hospital, Lahore, Pakistan; d Kabir Medical College, Gandhara University, Peshawar, Pakistan; e Dow University Hospital–Ojha Campus, Karachi, Pakistan; f Department of Diagnostic Imaging, Sidra Medicine, Doha, Qatar; g Department of Internal Medicine, Hamad Medical Corporation, Qatar

**Keywords:** CT scan, MRI, Radiology, Education, Radiation, Radiologist

## Abstract

Medical imaging techniques are a helpful tool for physicians to diagnose and treat diseases. Some of these techniques are conventional and include X-rays, Ultrasounds while others are advanced imaging modalities such as MRI and CT scans. Recently, more and more physicians are relying on these advanced imaging modalities because of advancements in technology, increased patient demand, greater finances, and the fear of any malpractice suits in case of missed diagnosis. While these techniques, no doubt, offer a quicker and correct diagnosis owing to their sharp resolution and sensitivity, they do expose the patient to a great source of radiation, are expensive, time consuming, and are not an ideal means to be used in all situations. Thus, it is crucial to mitigate their unnecessary use. The following article focuses on the growing use of such techniques, their advantages and disadvantages and how to alleviate their exaggerated use.

Medical imaging techniques aid in the diagnosis and treatment of both adult and children population [1], and lately there has been a rise in the use of medical imaging techniques for the purposes of diagnosis and follow up of diseases [2]. It is important to acknowledge the fact that where imaging facilities pave the way for diagnosis and treatment, they do have some drawbacks such as higher costs and disadvantages to patients in case of an incidental finding, aggressive diagnosis, unnecessary anxiety and, and radiation exposure [3].

The rise in the use of imaging facilities can be due to advancements in technology, increased demand by physicians and patients, and greater financial means [3]. There has been a rise of 8% use of computed tomography (CT) scans in the last decade or so [4]. A study showed that between 2001 and 2010 the rates of CT scans done in emergency department quadrupled for patients with respiratory symptoms but regarded such rise in the number of CT scans in emergency settings as useless [4]. In Ontario and 7 integrated healthcare systems of United States (US), use of CT, magnetic resonance imaging (MRI) and ultrasound have increased, with the greatest annual growth occurred between years 2000–2006 and sustained growth between 2012 and 2016 (1–5% annually) for almost all ages [3]. About 30% of imaging examination is deemed needless, and contributes $30 billion annually in US [3].

The advancement in technology has encouraged the physicians to order more CT scans and MRI for the conditions used to be investigated with more basic imaging techniques [5]. CT scan provides a better view of the pathologies than a plain radiograph. For instance, CT pulmonary angiography is the single best imaging technique to identify and follow the work up on pulmonary embolism due to its high resolution and fast speed, and thus summates the expenditure on chest imaging [5]. CT scan with its greater specificity and sensitivity and increased visualization ability can pick up small opacities missed on chest x-rays, and better view certain regions such as lung bases and lingula [10]. Enhanced resolution of advanced modalities also helps rule out the possibility of cancer in doubtful lesions, thus greater number of tests are being ordered [5]. Continued follow up in cases of cancer further adds to the number of imaging done [5]. Demands by patients and the pressure on the physicians to satisfy their demands and escape any malpractice lawsuit are also reasons for a rise in utility of advanced imaging [5]. Missed appendicitis was the most frequent reason for insurance of malpractice in 1984, and hence since then advanced imaging modalities have been routinely used for confirming the diagnosis of appendicitis [8]. Nature of the healthcare facility also determines the imaging advised [8]. Academic and urban hospitals are more likely to order advanced imaging modalities since patients are mostly under the care of trainee doctors [8]. The greater availability of CT scanners and MRI suits are also a contributor to the increased use of these imaging modalities [5]. One worthwhile reason of a rise in the use of advanced imaging is the fact that their use is often initiated long before data proves their significance and once, they have been incorporated into clinical practice, it is difficult to retract them [3]. Anatomic location of pathologies has also played a role in determining the imaging choices [5]. For example, the greatest rise in the investigations involving central nervous system, spine, chest, and cardiac system also contributes to a larger number of CT scans, and MR imaging being carried out [5].

It is important to realize that not every imaging modality is suitable for every situation [2]. Among the imaging modalities, ultrasound is one of the most readily available, inexpensive and patient-friendly techniques [2]. It does not accompany significant adverse effects and is safe to be used in pregnancy and pediatrics imaging [2]. Ultrasound with its non-invasiveness, increased sensitivity and advantage of being repeatable is becoming a fundamental component in emergency settings as well [6]. With ultrasound, diagnosis, even at bedside, can be made. Moreover, it prevents the exposure of the patient to ionizing radiation [6]. Although ultrasound and plain x-rays are readily available and cheaper modalities, often they are limited in their ability to pick up fine details. CT scan offers high resolution, thereby allowing details missed by an ultrasound or x-ray to be identified [7]. For example, in cases of bacterial pneumonia, where the presentation is highly suggestive but chest x-ray is unremarkable, CT scan can be done additionally [10]. However, it should not be the primary investigation, and the routine investigation remains a chest x-ray [10]. In addition to the higher costs of CT and greater time required to produce images, it also comes at a cost of exposure to ionizing radiations which causes damage to the person's DNA [1], creating potential for cancerous growth in such patients [7] especially in young population [8]. The radiation exposure of 10 mSv CT in a 25-year-old patient is linked with an induced cancer risk of 1 in 900 individuals and fatal cancer risk of 1 in 1800 individuals [8]. CT being one of the greatest sources of radiation exposure is responsible for up to 1–2% of cancers in the United Kingdom and United States [8]. Although a much slower rise in the use of CT scans than earlier in adults and, increased use of MRI in children and reduction in CT scans in children is a sign of progress [3], there is still a need to spread awareness regarding radiation exposure. A study regarding healthcare providers in the US showed that less than half of the radiologists and only 9% of physicians working in the emergency department knew the risk of cancer with the use of CT scans [9].

Contrast enhanced CT scans, although necessary in certain circumstances such as assessment of neoplasms, can potentiate certain adverse effects [2], such as allergies to the contrast, and contrast induced nephropathy [2]. Contrast enhanced CT is also contraindicated in patients with symptomatic hyperthyroidism [2]. MRI being a non-ionizing radiation modality allows soft tissue contrast resolution [8]. However, the scan timing is long, minimum 15 min and the machine is not available 24/7 [8]. Furthermore, MRI cannot be performed in patients with implants, devices and foreign bodies especially those close to eye, major vessels, or spinal cord [2].

Dependence on advanced imaging techniques in clinical practice is also impactful on education of the new generation. The new generation radiologists and physicians are less experienced in the use of x-rays and ultrasounds, and less confident in establishing the diagnosis of some conditions using those imaging modalities. According to the European Society of Radiology ultrasound subcommittee, the young radiologists often regard ultrasound as less appealing than CT or MRI for some reasons, like physical presence and full attention for a specific period of time, direct contact with the patients and perceived high patient turnover. The most important point is educating and preparing the new young radiologists about the use of newer ultrasound technological developments and their clinical applications [11].

It is important to perform the right imaging tests at the right times in order to establish the correct diagnosis and allow a prompt treatment and better patient care [4]. Not only should the rising number of the imaging tests be addressed, but also the safety level with which they operate [4]. Physicians shouldn't be entirely independent in ordering these tests [4]; radiologists should guide the physicians on the type of imaging necessary and how to reduce the radiation exposure while maintaining the authenticity of diagnosis [4]. Overuse or misuse of imaging techniques should be penalized [4]. Radiation protocols should be followed and how much radiation a patient receives from an imaging technique should be made evident [4]. Finances pertaining to imaging tests should be adjusted so as to improve the quality of these tests and not the quantity [4]. Measures like the Image Wisely Campaign by American College of Radiology that aims to curb unnecessary testing and reducing the radiation content exposed by the imaging should be taken [4]. Structured training and enriching the new generation physicians and radiologists experience on the classic imaging modalities should be emphasized in the training curricula. Avoiding unnecessary testing can prevent harm to the patients and have a positive impact on the finances and the overall clinical practice.

## Provenance and peer review

Externally peer reviewed not commissioned.

## Financial Support

No financial support was acquired for this article.

## Ethical approval

Not required.

## Please state any sources of funding for your research

All sources of funding should be declared as an acknowledgement at the end of the text. Authors should declare the role of study sponsors, if any, in the collection, analysis and interpretation of data; in the writing of the manuscript; and in the decision to submit the manuscript for publication. If the study sponsors had no such involvement, the authors should so state.

## Author contribution

M.J.T, S·Y and Z.Y conceived the idea; S·W, O.A, I·U, M.J.T, and Z.Y, collected the data; I·U, S·W, S.G.I, and M.J.T did write up of the manuscript; and finally, Z.Y, M.S.A, and S.G.I reviewed and revised the manuscript for intellectual content critically. All authors approved the final version of the manuscript.

## conflicts of interest

None.

## Consent

Not required.

## Registration of research studies

Name of the registry: Not required.

Unique Identifying number or registration ID: N/A.

Hyperlink to your specific registration (must be publicly accessible and will be checked):

## Guarantor

Muhammad Sohaib Asghar and Muhammad Junaid Tahir.

## Declaration of competing interest

The authors declare no conflict of interest.
